# Regulation of IL-17 in autoimmune diseases by transcriptional factors and microRNAs

**DOI:** 10.3389/fgene.2015.00236

**Published:** 2015-07-14

**Authors:** Deena Khan, S. Ansar Ahmed

**Affiliations:** Department of Biomedical Sciences and Pathobiology, Virginia–Maryland College of Veterinary Medicine, Virginia Polytechnic Institute and State UniversityBlacksburg, VA, USA

**Keywords:** interleukin 17, autoimmune, transcription, microRNA, inflammation

## Abstract

In recent years, IL-17A (IL-17), a pro-inflammatory cytokine, has received intense attention of researchers and clinicians alike with documented effects in inflammation and autoimmune diseases. IL-17 mobilizes, recruits and activates different cells to increase inflammation. Although protective in infections, overproduction of IL-17 promotes inflammation in autoimmune diseases such as multiple sclerosis, rheumatoid arthritis, psoriasis, among others. Regulating IL-17 levels or action by using IL-17-blocking antibodies or IL-17R antagonist has shown to attenuate experimental autoimmune diseases. It is now known that in addition to IL-17-specific transcription factor, RORγt, several other transcription factors and select microRNAs (miRNA) regulate IL-17. Given that miRNAs are dysregulated in autoimmune diseases, a better understanding of transcriptional factors and miRNA regulation of IL-17 expression and function will be essential for devising potential new therapies. In this review, we will overview IL-17 induction and function in relation to autoimmune diseases. In addition, current findings on transcriptional regulation of IL-17 induction and plausible interplay between IL-17 and miRNA in autoimmune diseases are highlighted.

## Introduction

Interleukin 17 (IL-17) was initially termed in Rouvier et al. (1993) as cytotoxic T lymphocyte-associated antigen-8 (CTLA-8) when it was first cloned from a rodent cDNA sequence. Subsequently, IL-17 was also identified in humans (Yao et al., 1995). Among the members of IL-17 family, IL-17A (hence referred as IL-17) and IL-17F are known for their important functional and biological properties. IL-17A and IL-17F are 50% homologous and map to the same chromosomal loci. They exist either as homodimers or IL-17A/F heterodimers (Liang et al., 2007). IL-17A is known to be secreted by many cell types including: CD4^+^ (Th17), CD8^+^ (Tc17), γδ^+^ T cells, natural killer cells, mast cells, neutrophils among other cell types (Rachitskaya et al., 2008; Lin et al., 2011; Zhao et al., 2012; Gelderblom et al., 2014; Li et al., 2014a; Ravichandran et al., 2014).

Although IL-17 is known to have protective effects in infections, increased IL-17 and/or aberrant responses to IL-17 has been shown to aggravate disease conditions and contribute to tissue injury as observed in many autoimmune diseases (Weaver and Murphy, 2007). IL-17 stimulates the production of various inflammatory mediators such as IL-6, IL-8, CNTF, TGF-β2, IL-10, BMP6, IL-1α, TNF-α, CCL19, CCL4, and M-CSF, CXCL1, CXCL2, CCL2, CCL12, CCL20 (Kang et al., 2010; Nardinocchi et al., 2014; Rodgers et al., 2014); MCP-1, KP, macrophage inflammatory protein (MIP)-2, TIMP-1, granulocyte chemotactic protein-2 (GCP-2) and matrix metalloproteinases (MMPs) -3, 9, and 13 (Qiu et al., 2009) and nitric oxide, HGF, MCP-1, KC, MIP-2, PGE1, PGE2, and VGEF (Numasaki et al., 2004). Studies have demonstrated that IL-17 cosynergizes with different ligands and signaling pathways such as toll-like receptor (TLR) ligands, B cell-activating factor (BAFF), IFNγ, IL-1β, CD40-ligand and TNFα to fine-tune inflammatory responses (Woltman et al., 2000; Andoh et al., 2001; Liu et al., 2014a; Nardinocchi et al., 2014; Francois et al., 2015).

## IL-17 and Autoimmune Diseases

In healthy homeostatic conditions, the levels of IL-17A in human sera are undetectable, however, the levels are markedly increased in inflammatory bowel disease, psoriasis, systemic lupus erythematosus (SLE), multiple sclerosis (MS), and rheumatoid arthritis (RA; Wang et al., 2012; Jiang et al., 2014; Babaloo et al., 2015; Mease, 2015; Talaat et al., 2015). IL-23 has been shown to be critical for expansion, and/or survival and stabilization of Th17 cells by activating STAT3 and partially STAT4 (Aggarwal et al., 2003; Harrington et al., 2005). Interaction of IL-23-producing APCs and Th17 cells has been shown to have a role in many autoimmune diseases. In support of this view, targeting IL-23 pathway, IL-17 production or action by using IL-17R antagonist and IL-17A-blocking antibodies have been shown to attenuate autoimmune diseases (Hueber et al., 2010; Yeilding et al., 2011; Leonardi et al., 2012; Papp et al., 2012; Sofen et al., 2014). In this regard, several clinical trials are underway to treat psoriasis. These include, ustekinumab anti-p40-IL-23 mAb and guselkumab, an anti-IL-23-specific mAb, ixekizumab and secukinumab (anti–IL-17A mAbs) and brodalumab (an anti-IL-17RA mAb) are currently under clinical trials. A recent study has demonstrated that a combination of inhibition of IL-23 and IL-17 is more efficacious in treating Th17-mediated autoimmunity in mouse models (Mangan et al., 2015). In addition, human recombinant IL-37 has also been shown to decrease IL-17 expression and Th17 cell frequency in PBMCs and CD4^+^ T cells from RA patients (Ye et al., 2015). Together these studies have shown promising results of targeting IL-17 induction and signaling pathways in the treatment of chronic autoimmune diseases.

## Molecular Aspects of IL-17 Induction

Although initial reports showed that TGFβ1 inhibits IL-17A production in a dose-dependent manner in human naïve CD4^+^ T cells (Acosta-Rodriguez et al., 2007), other studies have shown that low concentrations of TGFβ1 in combination with either IL-21 (Yang et al., 2008a), or IL-1β and IL-23 (Manel et al., 2008; Duhen and Campbell, 2014) or IL-1β, IL-23, and IL-6 (Volpe et al., 2008) promote differentiation of human CD4^+^ T cells into Th17 cells. Interestingly, it was recently shown that TGFβ3-induced Th17 cells have pathogenic effector signature when compared to TGFβ1-induced Th17 cells (Lee et al., 2012). Studies have also shown that IL-17 expression may be transient and not a terminal/end-stage Th cell differentiation (Kurschus et al., 2010; Hirota et al., 2011). Further, there is dynamic plasticity among Th subsets and Th17 differentiation is highly dependent on the kind of stimuli (polarizing conditions) received from the local tissues.

**FIGURE 1:**
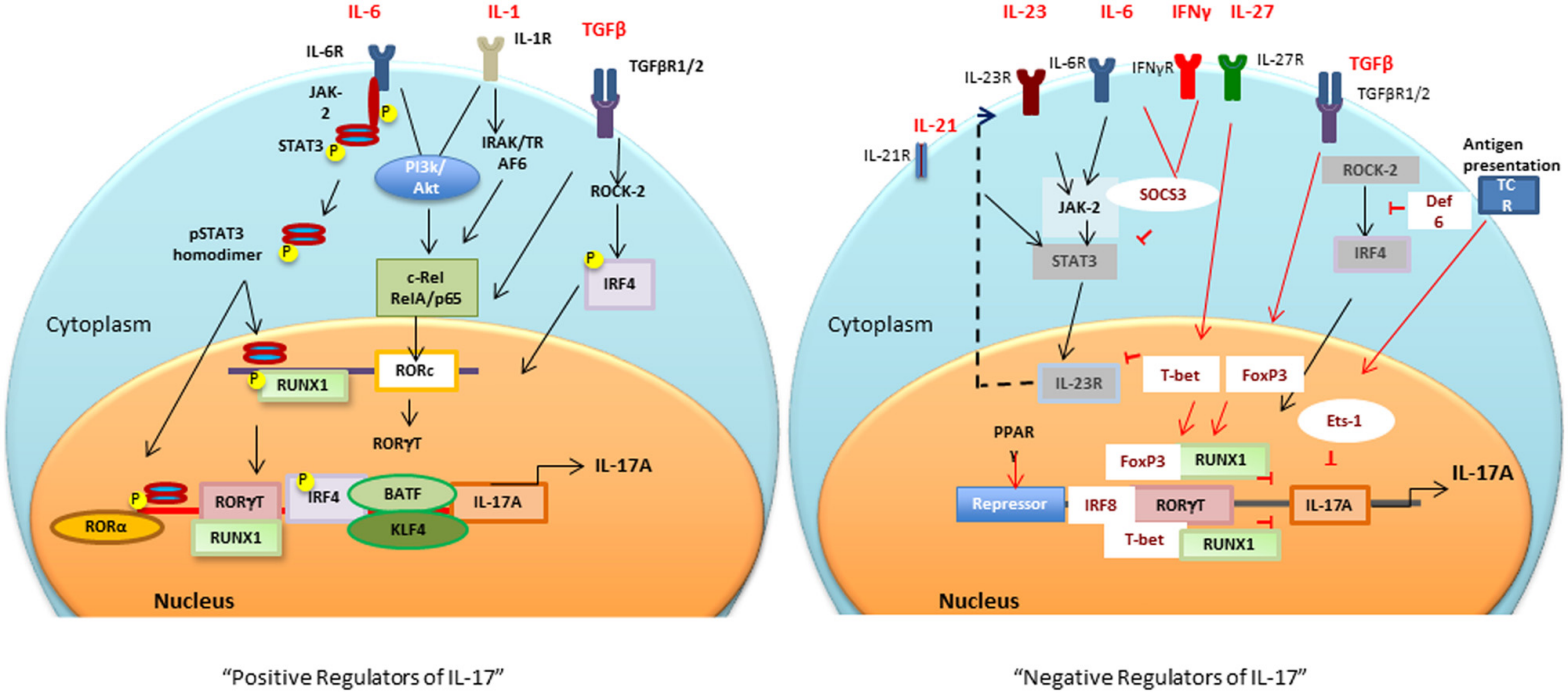
**Positive and negative transcriptional tegulators of IL-17 induction: Different cytokines and antigen specific stimuli trigger (black arrows and lines) different signaling cascades for activation of *RORc* and consequently *Il17* gene.** Negative regulators (red arrows and lines), T-bet or FoxP3 interaction with RUNX1 prevents RORγt-RUNX1 interaction, which prevents RORγt-mediated IL-17 induction. Def6 binding to IRF4 prevents ROCK2-mediated IRF4 phosphorylation and subsequent IL-17 induction. PPARγ, peroxisome proliferator activated receptor γ; SOCS, suppressors of cytokine signaling; TCR, T cell receptor; BATF, B cell-activating transcription factor; IL, interleukin; TGFβ, transforming growth factor β; RORγt, retinoic acid-related orphan receptor γt; STAT, signal transducer and activator of transcription; IRF-4, interferon-inducible factor-4; RUNX1, Runt-related transcription factor 1; IRAK, IL-1 receptor-associated kinase; TRAF6, TNF receptor associated factor-6; ROCK, Rho-associated serine/threonine kinases.

Sentinel cells of innate immune system (neutrophils, γδ T, Lti, Paneth, and iNKT cells) also secrete IL-17 (Cua and Tato, 2010). These cells constitutively express transcriptional regulators for IL-17 induction (discussed in the next section), therefore, upon activation produce IL-17 within hours of stimuli (Sutton et al., 2009; Cua and Tato, 2010). The next section will focus on multiple transcription factors involved in the positive and negative regulation of IL-17 (**Figure 1**).

### Positive IL-17 Regulators

#### ROR*γ*t and RUNX1

It is now well established that for Th17 differentiation, it is critical to have TGFβ1 in the presence of IL-1, IL-6, or IL-21 to decrease suppressive FoxP3 and upregulate *RORc* gene encoded unique lineage-specific transcription factor, RORγt, an retinoic acid related-orphan nuclear receptor (Bettelli et al., 2006; Ivanov et al., 2006; McGeachy et al., 2007; Yang et al., 2008b; Biswas et al., 2010; Ikeda et al., 2014). Runt-related transcription factor 1 (Runx1) regulates Th17 differentiation by upregulating RORγt expression and by direct binding to RORγt (Zhang et al., 2008; Liu et al., 2015). Interestingly, a study has shown that IL-17-secreting Treg cells (Tr17) have co-expression of FoxP3, RORγt, Runx1, and Runx3 (Li et al., 2012). A recent study has shown that RORγt-transcriptional activity, and subsequent IL-17 induction is increased by Sirtuin 1 (SIRT1), a protein deacetylase. Inhibition of SIRT1 by chemical Ex-527 based inhibition or T cell specific deletion strongly suppressed the development of experimental autoimmune encephalitis (EAE) in mice (Lim et al., 2015). In addition, a selective RORγt inverse agonist (TMP778) has been shown to inhibit Th17 signature gene expression, and IL-17 production from Tc17 and γδ T cells indicating the therapeutic potential of targeting RORγt in inflammatory conditions (Skepner et al., 2014).

#### STAT3

Activation of IL-6R (ligand binding IL-6Rα and signal transducing gp130) by IL-6 results in activation of JAK-2/STAT3 pathway. Activated STAT3 binds to the promoter of IL-17A and IL-17F (Chen et al., 2006). Depletion of either STAT3 or gp130 in CD4^+^ T cells by utilizing Cre-loxP recombination results in decreased RORγt expression and Th17 differentiation, suggesting that IL-6-gp130-STAT3 regulate IL-17 induction at least in part by regulating RORγt levels (Nishihara et al., 2007). *In vivo* inhibition of JAK2-STAT3 pathway by AG490 was recently shown to decrease Th17 but increase Tregs in collagen-induced arthritis mice (Park et al., 2014).

#### NF-κB

It has recently been shown that members of NF-κB family RelA (p65) and c-Rel bind to promoters of RORγ and RORγt, respectively (Ruan et al., 2011). The positive role of NF-κB in IL-17 induction was further substantiated by the findings that activation of NF-κB increases secretion of IL-17 (Huang et al., 2008). c-Rel deficient mice have decreased EAE due to impaired activation of *RORc* gene and subsequently decreased Th17 development (Lazarevic et al., 2011). Peripheral blood mononuclear cells (PBMCs) from RA patients have increased IL-17 induction by activation of PI3K/Akt, which increases the DNA binding activity of NF-κB (Kim et al., 2005). In addition, in DC and CD4 T cells co-culture system, dendritic cells (DCs) that are deficient in RelB have decreased induction of IL-12p70, IL-23, and IL-6 when compared to control DCs, thereby resulting in decreased Th17- and Th1-related markers but increased Th2 and Treg markers (Yang et al., 2010).

#### Interferon Regulatory Factor 4

Interferon regulatory factor 4 (IRF4) is also critical for IL-17 and IL-21 induction (Ciofani et al., 2012; Huber and Lohoff, 2014). It has been shown that IRF4-deficient mice have decreased RORα and RORγt expression but increased FoxP3 levels (Brustle et al., 2007; Huber et al., 2008). TGFβ-mediated activation of Rho-ROCK pathway, promotes phosphorylation of IRF4 by ROCK kinase. Once phosphorylated, IRF4 translocates to nuclei and binds to IL-17 and IL-21 promoters (Biswas et al., 2010; Mudter et al., 2011). It is noteworthy that in autoimmune models such as MRL/lpr, there is enhanced ROCK2 activation concomitant with increased IRF4 function and IL-17 levels (Biswas et al., 2010).

#### Other IL-17 Promoting Transcription Factors

Kruppel-like factor (KLF) 4, has been shown to regulate Th17 development by binding to the IL-17 promoter directly without altering RORγt expression (Lebson et al., 2010; An et al., 2011). Basic leucine zipper transcription factor, ATF-like (BATF), synergizes with RORγt to induce IL-17 by direct interaction with conserved intergenic elements of *Il17A/Il17F* loci (Jordan-Williams et al., 2013). Additionally, other signaling pathway Rho-associated kinase, ROCK2 (Zanin-Zhorov and Waksal, 2015), Ets-family transcription factor (Etv5; Pham et al., 2014), Sphingosine 1-phosphate- /type 1 S1P receptors (S1P_1_s; Liao et al., 2007), RORα (Yang et al., 2008b) and aryl hydrocarbon receptor (Hayes et al., 2014) have also been shown to be important for the induction of IL-17.

### Negative IL-17 Regulators

Both IFNγ and IL-4 markedly inhibit Th17 differentiation (Harrington et al., 2005; Yeh et al., 2014). In addition, IL-25 and IL-27 have been found to negatively regulate Th17 cells (Batten et al., 2006; Kleinschek et al., 2007; Tang et al., 2015). Suppressor of cytokine signaling (SOCS3) also negatively regulates IL-6-gp160 signal transduction resulting in decreased IL-17 (Babon et al., 2014; Vartoukian et al., 2014). FoxP3 interacts directly with RORγt through the exon 2 region and forkhead domain of FoxP3 and suppresses the activation of the IL-17 promoter (Ichiyama et al., 2008). Moreover, it has also been shown that Runx1 interacts with FoxP3 and negatively regulates Th17 differentiation (Zhang et al., 2008). T-bet, a Th1-lineage specific transcription factor, also suppresses Th17 development by binding to Runx1 via tyrosine 304 of T-bet. This T-bet-Runx1 binding has been shown to block the transactivation of *RORc* gene and therefore IL-17 induction (Lazarevic et al., 2011). These studies indicate that Runx1 associates either with RORγt to upregulate IL-17, or with T-bet /FoxP3 to suppress Th17 differentiation (Gocke et al., 2007). Interestingly, a recent report has shown that in EAE, pathogenic IFNγ-secreting Th17 cells have co-expression of T-bet, Runx-1 or -3 (Wang et al., 2014). These findings suggest a critical role of Runx1 in differentiation of Th cells into different specific Th cell lineages.

Another T-bet interacting transcription factor, v-ets erythroblastosis virus E26 oncogene homolog 1 (ETS-1), has been shown to inhibit Th17 differentiation. ETS-1-deficient mice have increased IL-17 levels (without affecting RORγt), suggesting that ETS-1 is a negative regulator of IL-17 (Moisan et al., 2007). Recently, IRF8, also known as ICSBP, has been shown to physically interact with RORγt and regulate IL-17 by silencing Th17 differentiation and downregulating Th17-associated genes (Qi et al., 2009; Ouyang et al., 2011). Peroxisome proliferator-activated receptor γ (PPARγ) also acts as an intrinsic suppressor of Th17. It prevents the removal of repressor complexes from RORγt promoter, resulting in decreased RORγt expression and RORγt-induced Th17 differentiation (Klotz et al., 2009). In addition, there are other compounds such as digoxin and signaling molecules [e.g., STAT1, STAT4, STAT5, STAT6, NFAT, SOCS1, early growth response gene 2 (Egr-2), IRF-4 binding protein Def6, epidermal fatty acid binding protein (E-FABP)] which negatively regulate IL-17 induction (Fujita-Sato et al., 2011; Huh et al., 2011; Grange et al., 2013; Miao et al., 2013). Overall these reports confirm that there are multiple transcription factors, which fine-tune and tightly regulate IL-17 induction. In the next section, we will focus on the miRNAs, which epigenetically either regulate IL-17 induction or response in different autoimmune and other disease conditions.

## IL-17 and miRNA

Positive correlations in miRNA expression and IL-17 levels have been observed in different studies (**Table 1**). In experimental autoimmune uveoretinitis (EAU), miR-142-5p and miR-21 were increased but miR-182 decreased in eye. The kinetic changes in these miRNA paralleled with increased IL-17 levels (Ishida et al., 2011). Given that IL-17 levels are increased in skin lesions and sera of psoriasis vulgaris patients, it is noteworthy that miR-1266 levels, a putative regulator of IL-17A, were also increased in the sera of these patients (Ichihara et al., 2012). miR-146 is also positively correlated with IL-17A levels in psoriasis (Xia et al., 2012) and in RA disease severity, and is co-expressed with IL-17A in the PBMC and synovium in RA patients (Niimoto et al., 2010). Inverse relationship between select miRNAs and IL-17 has also been observed in autoimmune conditions and other diseases (**Table 1**). For example, combined treatment with anti-TNFα/disease-modifying antirheumatic drugs (anti-TNFα/DMARDs) increased miR-16-5p, miR-23-3p, miR125b-5p, miR-126-3p, miRN-146a-5p, miR-223-3pmiRNA with a concomitant decrease in TNFα, interleukin (IL)-6, IL-17, rheumatoid factor (RF), and C-reactive protein (CRP; Castro-Villegas et al., 2015). Imbalance of Th17/Treg in RA patients has been associated with decreased miR-21 levels, increased STAT3 activation and decreased STAT5/pSTAT5 protein and FoxP3 mRNA levels (Dong et al., 2014). In experimental autoimmune myasthenia gravis (EAMG), there is marked downregulation of miR-145 expression. Administration of lentiviral-miR-145 decreased EAMG disease severity by decreased IL-17 production (Wang et al., 2013). miRNA dysregulation has been best studied in cancer and in several cancer studies miRNA regulation of IL-17 has been demonstrated, which may have implications for autoimmune diseases (Matsuyama et al., 2011; Arisawa et al., 2012; Zhang et al., 2013; Li et al., 2014b). A positive correlation in expession of miR-133b and miR-206 and IL-17 in both αβ and γδ T cells in human and inbred mouse strains have also been reported (Haas et al., 2011). It was found that these miRNAs were clustered nearly 45 kb upstream of *Il17a/f* locus (Haas et al., 2011). On the other hand, in dermatomyositis patients, there is downregulation of miR-206 and upregulation of miR-206 predicted target KLF4, a positive regulator of IL-17 and Th17 cells (Tang et al., 2015). It is likely that miR-206 regulation of IL-17 may be context dependent.

**Table 1:** miRNAs involved in regulation of IL-17 induction/response.

miRNA	IL-17 correlation	Signaling pathway	Autoimmune/infection	Reference
lmiR-142-5p	Positive	-	Experimental autoimmune uveoretinitis	Ishida et al. (2011)
miR-21	Positive		uveoretinitis	
miR-182	Negative			
lmiR-1266	Positive		Psoriasis	Ichihara et al. (2012)
lmiR-146	Positive		Psoriasis RA	Xia et al. (2012) Niimoto et al. (2010)
lmiR-29a	Positive		Tuberculosis	Kleinsteuber et al. (2013)
lmiR-21	Negative	STAT3	RA	Dong et al. (2014)
lmiR-15a/16, miR-34a, miR-194	Negative		Multiple myeloma	Li et al. (2014b)
lmiR-135b	Positive	STAT6 GATA3	Anaplastic large cell lymphoma	Matsuyama et al. (2011)
lLet-7e	Negative		Liver disease	Zhang et al. (2013)
lLet-7f	Negative	IL-23R		Li et al. (2011)
lmiR-145	Negative		Experimental autoimmune myasthenia gravis	Wang et al. (2013)
lmiR-223	Positive	Roquin	Colonic inflammation	Schaefer et al. (2011)
lmiR-146b miR-21	Positive	RORγt SMAD7	Viral myocarditis EAE	Liu et al. (2013) Murugaiyan et al. (2015)
lmiR-155	Positive	SOCS1	*Helicobacter pylori Streptococcus pneumoniae* RA	Oertli et al. (2011) Verschoor et al. (2014) O’Connell et al. (2010) Yao et al. (2011)
lmiR-212	Positive	Bcl6		Nakahama et al. (2013)
lmiR-206	Positive Negative	KLF4	Dermatomyositis	Haas et al. (2011) Tang et al. (2015)
lmiR-132	Negative		EAE	Hanieh and Alzahrani (2013)
lmiR-23b	Negative	TAB2, TAB3 IKK-α	EAE Bechet’s disease	Zhu et al. (2012) Qi et al. (2014)
lmiR-20b	Negative	RORγt STAT3	MS/EAE	Zhu et al. (2014)
lmiR-873	Positive	A20 NF-κB	MS/EAE	Liu et al. (2014b)
lmiR-326	Positive	Ets-1	MS/EAE	Du et al. (2009)

A study demonstrated that *in vitro* treatment of colonic intraepithelial lymphocyte with IL-10 decreased miR-19a, miR-21, miR-31, miR-101, miR-223, and miR-155 and IL-17 (Schaefer et al., 2011). miR-223 affects IL-17 by targeting Roquin, which resulted in increased IL-17 expression (Schaefer et al., 2011). In PPARγ deficient mice, there is increased colonic inflammation accompanied with increased IL-17A and miR-146b expression (Viladomiu et al., 2012).

A recent report has shown that miR-21 increased Th17 differentiation by targeting and depleting a negative regulator of TGF-β signaling SMAD-7 (Murugaiyan et al., 2015). Treatment of wild type mice with anti miR-21 oligonucleotide decreased EAE clinical severity along with decreased Th17 cells (Murugaiyan et al., 2015). In MS patients, there is downregulation of miR-20b. In EAE, miR-20b overexpression decreased disease severity by decreasing Th17 differentiation by targeting RORγt and STAT3 (Zhu et al., 2014). There is upregulation of miR-873 in brain tissue of EAE mice and in IL-17 activated mouse primary astrocytes (Liu et al., 2014b). In EAE model, miR-873 induced by IL-17 stimulation aggravated disease severity and increased inflammation by targeting A20/NF-κ (Liu et al., 2014b). Importantly, Du et al. (2009) reported that miR-326 expression correlated with MS disease severity in human patients. Further in EAE mice, miR-326 played an important role in pathogenesis by regulating Th-17 cell differentiation through translational inhibition of Ets-1, a negative regulator of Th17 differentiation (). In MS patients there is decreased expression of an IL-6-related miRNA, miR-26a (Zhang et al., 2015). *In vivo* silencing of miR-26a increased Th17-related cytokines and EAE severity (Zhang et al., 2015).

miR-155 deficiency results in decreased severity of different autoimmune diseases such as EAE, collagen induced arthritis (CIA) by impairment of Th17 polarization and decreased IL-17 levels (O’Connell et al., 2010; Bluml et al., 2011; Murugaiyan et al., 2011). The above studies indicate a strong correlation between miR-155 expression and Th17 differentiation, which is potentially mediated by miR-155 targeting of signaling molecule, SOCS1 (Yao et al., 2011, 2012).

Interleukin 17 has been shown to down regulate miR-23b expression in human fibroblast-like synoviocytes, mouse primary kidney cells and astrocytes. miR-23b suppresses IL-17-mediated autoimmune inflammation by targeting TNF-α- or IL-1β-induced NF-κB activation by targeting TGF-β-activated kinase 1/MAP3K7 binding protein 2 (TAB2), TAB3 and inhibitor of NF-κB kinase subunit α (IKK-α; Zhu et al., 2012). Behcet’s disease (BD) patients have increased activation of Notch pathway and Th17 response but decreased miR-23b (Qi et al., 2014). These studies indicate the potential of miR-23b as a therapeutic target for IL-17-related autoimmune conditions. miR-21 levels are also increased in BD patients and decrease in miR-21 in herpes simplex virus (HSV)-induced BD mouse model decreased serum IL-6 and IL-17 levels and improved disease condition (Choi et al., 2015).

AHR activation results in upregulation of miR-132/212 cluster under Th17 inducing conditions (Nakahama et al., 2013). However, overexpression of miR-132 in CD4 T cells from EAE mice decreased IL-17 and IFNγ and T cell proliferation (Hanieh and Alzahrani, 2013). Interestingly, miR-212 targeted B-cell lymphoma 6, a negative regulator of Th17 differentiation (Nakahama et al., 2013). These findings suggest that miRNA regulation and correlation with IL-17 is dependent on disease model.

Taken together, these studies indicate that IL-17 is regulated by interplay of multiple transcription factors and miRNAs and vary with different disease condition and cell type studied. It is recognized that in addition to miRNAs, other epigenetic mechanisms such as histone modifications and DNA methylation also contribute to autoimmune diseases. However, this topic is beyond the scope of this concise focused review.

## Concluding Comments

Overall, IL-17 is regarded as a potent proinflammatory cytokine that is essential for defense against pathogens. However, dysregulated IL-17 production or response has been associated with tissue damage in various inflammatory diseases. Given that IL-17 has now been associated with many inflammatory and autoimmune diseases, a better understanding of IL-17 induction and regulation is imperative to institute novel effective targeted therapeutic strategies. While RORγt, is considered as a IL-17-specific transcription factor, recent collective data clearly show that induction of IL-17 is regulated by multiple transcription factors. Transcription factors that positively and negatively regulate IL-17 have now been identified. In addition, post-transcriptional regulation of IL-17 by specific miRNAs is now increasingly appreciated. Aberrant miRNA expression is reported in several human autoimmune diseases (Dai and Ahmed, 2011; Dai et al., 2013; Khan et al., 2015). Dysregulated miRNA expression profiles have the potential to serve as good diagnostic and prognostic marker and/or therapeutic targets. Signature miRNA profile can be potentially used as novel biomarkers for Th17-mediated immune reactions. However, more in-depth and mechanistic studies are required to further define the role of miRNAs in IL-17 induction and interplay of miRNA with IL-17-related transcription factors and signaling pathways. Since blocking a major cytokine such as IL-17 may have unintended consequences, more refined (tissue-dependent) manipulation of select IL-17-regulating-miRNAs may be a viable therapeutic option in some diseases.

## Author Contributions

DK and SA designed the work, drafted and revised the work and finally approved the version to be published and agree to be accountable for all aspects of the work.

## Conflict of Interest Statement

The authors declare that the research was conducted in the absence of any commercial or financial relationships that could be construed as a potential conflict of interest.
